# A salen-type trinuclear Zn_2_Gd complex

**DOI:** 10.1107/S160053681204634X

**Published:** 2012-11-17

**Authors:** Yong-Mei Tian, Hong-Feng Li, Bing-Lu Han, Qian Zhang, Wen-Bin Sun

**Affiliations:** a Key Laboratory of Functional Inorganic Material Chemistry (HLJU), Ministry of Education, School of Chemistry and Materials Science, Heilongjiang University, Harbin 150080, People’s Republic of China

## Abstract

In the trinuclear title complex, di-μ-acetato-1:2κ^2^ *O*:*O*′;2:3κ^2^ *O*:*O*′-bis­{μ-6,6′-dimeth­oxy-2,2′-[cyclo­hexane-1,2-diylbis(methanylyl­idene)]diphenolato}-1:2κ^6^ *O* ^1^,*N*,*N*′,*O* ^1′^:*O* ^6^,*O* ^6′^;2:3κ^6^ *O* ^6^,*O* ^6′^:*O* ^1^,*N*,*N*′,*O* ^1′^-2-gadolinium(III)-1,3-dizinc hexa­fluor­idophosphate methanol monosolvate monohydrate, [GdZn_2_(C_22_H_24_N_2_O_4_)_2_(CH_3_COO)_2_]PF_6_·CH_3_OH·H_2_O, the two Zn^II^ ions are located in the inner N_2_O_2_ cavities of two 6,6′-dimeth­oxy-2,2′-[cyclo­hexane-1,2-diylbis(methanylyl­idene)]diphenolate (*L*) ligands. Both Zn^II^ ions are five-coordinated by two O atoms and two N atoms from the *L* ligand and one O atom of an acetic acid mol­ecule, giving rise to a square-pyramidal geometry around the Zn^II^ ions. The Gd^III^ ion is nine-coordinated by four O atoms from the outer O_2_O_2_ sites of one ligand, and three O atoms from another ligand, in which there is one non-coordinating meth­oxy O atom. Two further O atom from different acetate ligands complete the nine-coordinate environment.

## Related literature

For crystal structures of similar trinuclear complexes of salen-type Schiff base ligands, see: Wong *et al.* (2006 ▶); Wang *et al.* (2009 ▶).
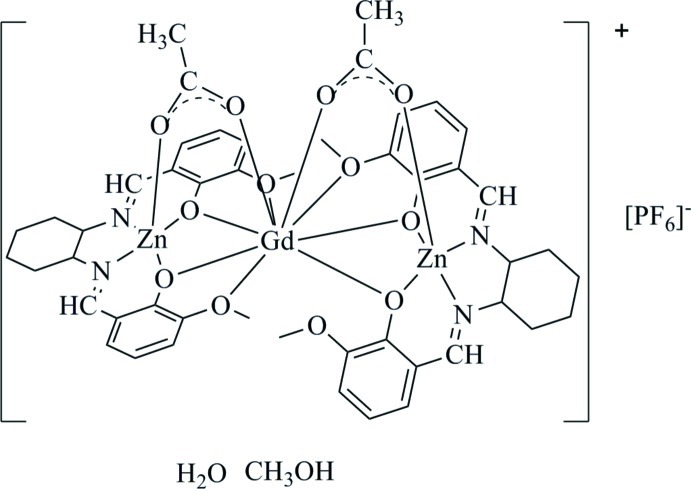


## Experimental

### 

#### Crystal data

[GdZn_2_(C_22_H_24_N_2_O_4_)_2_(C_2_H_3_O_2_)_2_]PF_6_·CH_4_O·H_2_O
*M* *_r_* = 1361.97
Monoclinic, 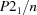

*a* = 15.8127 (12) Å
*b* = 20.6405 (16) Å
*c* = 18.5036 (14) Å
β = 114.369 (1)°
*V* = 5501.2 (7) Å^3^
*Z* = 4
Mo *K*α radiation
μ = 2.17 mm^−1^
*T* = 296 K
0.20 × 0.18 × 0.16 mm

#### Data collection

Rigaku R-AXIS RAPID diffractometer
Absorption correction: multi-scan (*ABSCOR*; Higashi, 1995 ▶) *T*_min_ = 0.671, *T*_max_ = 0.723
39974 measured reflections
13701 independent reflections
6858 reflections with *I* > 2σ(*I*)
*R*_int_ = 0.066

#### Refinement

*R*[*F* ^2^ > 2σ(*F* ^2^)] = 0.053
*wR*(*F* ^2^) = 0.157
*S* = 0.98
13701 reflections
695 parameters
19 restraints
H-atom parameters constrained
Δρ_max_ = 1.02 e Å^−3^
Δρ_min_ = −0.71 e Å^−3^

### 

Data collection: *RAPID-AUTO* (Rigaku, 1998 ▶); cell refinement: *RAPID-AUTO*; data reduction: *CrystalStructure* (Rigaku/MSC, 2002 ▶); program(s) used to solve structure: *SHELXS97* (Sheldrick, 2008 ▶); program(s) used to refine structure: *SHELXL97* (Sheldrick, 2008 ▶); molecular graphics: *SHELXTL* (Sheldrick, 2008 ▶); software used to prepare material for publication: *SHELXTL*.

## Supplementary Material

Crystal structure: contains datablock(s) I, global. DOI: 10.1107/S160053681204634X/pv2601sup1.cif

Structure factors: contains datablock(s) I. DOI: 10.1107/S160053681204634X/pv2601Isup2.hkl

Additional supplementary materials:  crystallographic information; 3D view; checkCIF report

## Figures and Tables

**Fig. 1.**
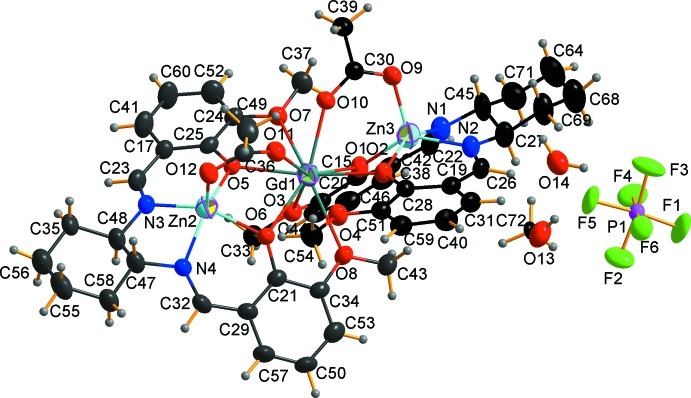
The molecular structure of the title compound showing the atomic numbering and 30% probability displacement ellipsoids.

## supplementary crystallographic information

### Comment

The Schiff base heteronuclear M—Ln complexes (*M* = transition metal and Ln = rare earth) have attracted much attention for their magnetic properties. Several mutinuclear complexes are reported that have been derived from aromatic salen type Schiff base ligands (Wong *et al.*, 2006; Wang *et al.*, 2009). In this article, we report the preparation and crystal structure of a Zn—Gd—Zn trinuclear coordination complex of the ligand *N*,*N*'-bis(3-methoxysalicylidene)propane-1,2-cyclohexanediamine.

The hexadentate Schiff base ligand (*L*) links Zn and Gd atoms into a trinuclear complex (Fig. 1). The Gd^III^ centre in the complex is nine-coordinated by seven oxygen atoms from ligand *L* and two oxygen atoms from two acetic acid molecules. The Zn^II^ center is five-coordinated by two nitrogen atoms and two oxygen atoms from the ligand and one oxygen atom from acetic acid and result in a square-pyramidal coordination geometry.

### Experimental

To a 1:1 MeOH/CH_2_Cl_2_ solution (20 ml) of the Schiff ligand (*L*) (0.386 g, 1.0 mmol) was slowly added a solid [Dy(acac)_3_H_2_O; acac = acetylacetone] (0.491 g, 1.0 mmol), after stirring for 10 minutes solid [Zn(CH_3_COO)_2_H_2_O] (0.220 g, 1.0 mmol) and NH_4_PF_6_ (0.164 g, 1.0 mmol) were added after refluxing and stirring for 1 h, at ambient temperature. After stirring for 5 h, the title complex as yellow solid was collected by filtration and was washed with MeOH; yield = 0.952 g (68%). Single crystals suitable for X-ray determination were obtained by slow diffusion of diethylether into a methanol solution of the powder sample over one week.

### Refinement

H atoms bound to C atoms were placed in calculated positions and treated as riding on their parent atoms, with C—H = 0.93, 0.96, 0.97 and 0.98 Å, for aryl, methyl, methylene and methyne H-atoms, respectively. The *U*_iso_(H) were allowed at 1.5*U*_eq_(C methyl) or 1.2*U*_eq_(C non-methyl). The hydroxyl and water H-atoms were located from a difference map and were included in the refinement with O—H = 0.85 Å and *U*_iso_(H) = 1.2*U*_eq_(hydroxy O) or 1.5*U*_eq_(water O). The command 'ISOR 0.01 013 014 c72' was used to restrain the ADPs and the distance between O13 and C72 was restrain at 1.50(0.01) Å.

### Crystal data

**Table:**

[GdZn_2_(C_22_H_24_N_2_O_4_)_2_(C_2_H_3_O_2_)_2_]PF_6_·CH_4_O·H_2_O	*F*(000) = 2748
*M**_r_* = 1361.97	*D*_x_ = 1.644 Mg m^−^^3^
Monoclinic, *P*2_1_/*n*	Mo *K*α radiation, λ = 0.71073 Å
Hall symbol: -p 2yn	Cell parameters from 25271 reflections
*a* = 15.8127 (12) Å	θ = 3.0–27.5°
*b* = 20.6405 (16) Å	µ = 2.17 mm^−^^1^
*c* = 18.5036 (14) Å	*T* = 296 K
β = 114.369 (1)°	Prism, yellow
*V* = 5501.2 (7) Å^3^	0.20 × 0.18 × 0.16 mm
*Z* = 4	

### Data collection

**Table:**

Rigaku R-AXIS RAPID diffractometer	13701 independent reflections
Radiation source: fine-focus sealed tube	6858 reflections with *I* > 2σ(*I*)
Graphite monochromator	*R*_int_ = 0.066
Detector resolution: 10.000 pixels mm^-1^	θ_max_ = 28.4°, θ_min_ = 1.4°
ω scans	*h* = −21→21
Absorption correction: multi-scan (*ABSCOR*; Higashi, 1995)	*k* = −27→27
*T*_min_ = 0.671, *T*_max_ = 0.723	*l* = −24→15
39974 measured reflections	

### Refinement

**Table:**

Refinement on *F*^2^	Primary atom site location: structure-invariant direct methods
Least-squares matrix: full	Secondary atom site location: difference Fourier map
*R*[*F*^2^ > 2σ(*F*^2^)] = 0.053	Hydrogen site location: inferred from neighbouring sites
*wR*(*F*^2^) = 0.157	H-atom parameters constrained
*S* = 0.98	*w* = 1/[σ^2^(*F*_o_^2^) + (0.0722*P*)^2^] where *P* = (*F*_o_^2^ + 2*F*_c_^2^)/3
13701 reflections	(Δ/σ)_max_ = 0.002
695 parameters	Δρ_max_ = 1.02 e Å^−^^3^
19 restraints	Δρ_min_ = −0.71 e Å^−^^3^

### Special details

**Table:**

Experimental. Analysis calculated for C_49_H_60_C_l0_F_6_GdN_4_O_14_PZn_2_: C, 42.11; H, 4.33; N, 4.01; found: C, 42.01; H, 4.20; N, 3.93%.
Geometry. All e.s.d.'s (except the e.s.d. in the dihedral angle between two l.s. planes) are estimated using the full covariance matrix. The cell e.s.d.'s are taken into account individually in the estimation of e.s.d.'s in distances, angles and torsion angles; correlations between e.s.d.'s in cell parameters are only used when they are defined by crystal symmetry. An approximate (isotropic) treatment of cell e.s.d.'s is used for estimating e.s.d.'s involving l.s. planes.
Refinement. Refinement of *F*^2^ against ALL reflections. The weighted *R*-factor *wR* and goodness of fit *S* are based on *F*^2^, conventional *R*-factors *R* are based on *F*, with *F* set to zero for negative *F*^2^. The threshold expression of *F*^2^ > σ(*F*^2^) is used only for calculating *R*-factors(gt) *etc*. and is not relevant to the choice of reflections for refinement. *R*-factors based on *F*^2^ are statistically about twice as large as those based on *F*, and *R*- factors based on ALL data will be even larger.

### Fractional atomic coordinates and isotropic or equivalent isotropic displacement parameters (Å^2^)

**Table:**

	*x*	*y*	*z*	*U*_iso_*/*U*_eq_	
C15	0.8093 (5)	0.5417 (3)	0.8460 (4)	0.0542 (15)	
C17	1.0251 (4)	0.5705 (3)	0.6400 (4)	0.0572 (16)	
C19	0.5217 (4)	0.3534 (3)	0.6962 (4)	0.0623 (17)	
C20	0.9052 (5)	0.5368 (3)	0.8676 (4)	0.0579 (16)	
C21	0.9314 (4)	0.3318 (3)	0.7457 (4)	0.0562 (16)	
C22	0.6815 (6)	0.5833 (3)	0.8827 (5)	0.072 (2)	
H22A	0.6689	0.6055	0.9209	0.086*	
C23	1.0793 (5)	0.5207 (3)	0.6260 (4)	0.0678 (19)	
H23A	1.1348	0.5343	0.6249	0.081*	
C24	0.9029 (4)	0.6117 (3)	0.6749 (4)	0.0566 (16)	
C25	0.9460 (4)	0.5589 (3)	0.6535 (4)	0.0511 (15)	
C26	0.4880 (4)	0.3973 (4)	0.7390 (4)	0.0695 (19)	
H26A	0.4456	0.3807	0.7572	0.083*	
C27	0.4768 (6)	0.4935 (4)	0.8043 (6)	0.091 (3)	
H27A	0.5041	0.4747	0.8577	0.110*	
C28	0.5987 (4)	0.3649 (3)	0.6782 (4)	0.0534 (15)	
C29	0.9993 (5)	0.2873 (3)	0.7438 (4)	0.0678 (19)	
C30	0.5880 (4)	0.5536 (3)	0.5878 (4)	0.0557 (16)	
C31	0.4745 (5)	0.2935 (3)	0.6750 (5)	0.075 (2)	
H31A	0.4245	0.2855	0.6878	0.091*	
C32	1.0496 (5)	0.2956 (3)	0.6943 (5)	0.077 (2)	
H32A	1.0969	0.2661	0.7013	0.092*	
C33	1.0239 (5)	0.4909 (4)	0.8334 (4)	0.080 (2)	
H33A	1.0610	0.5090	0.8846	0.119*	
H33B	1.0375	0.5128	0.7937	0.119*	
H33C	1.0376	0.4456	0.8333	0.119*	
C34	0.8923 (4)	0.3213 (3)	0.8004 (4)	0.0589 (16)	
C35	1.1788 (5)	0.4331 (4)	0.5599 (5)	0.079 (2)	
H35A	1.1320	0.4382	0.5062	0.094*	
H35B	1.2102	0.4743	0.5769	0.094*	
C36	0.7696 (4)	0.4078 (3)	0.4995 (4)	0.0546 (16)	
C37	0.7737 (5)	0.6472 (3)	0.6979 (5)	0.077 (2)	
H37A	0.8047	0.6874	0.6987	0.116*	
H37B	0.7660	0.6425	0.7464	0.116*	
H37C	0.7140	0.6471	0.6539	0.116*	
C38	0.8443 (6)	0.6068 (3)	0.9634 (4)	0.075 (2)	
H38A	0.8246	0.6299	0.9968	0.091*	
C39	0.5368 (5)	0.5861 (3)	0.5091 (4)	0.075 (2)	
H39A	0.4794	0.6035	0.5068	0.112*	
H39B	0.5239	0.5551	0.4673	0.112*	
H39C	0.5741	0.6205	0.5030	0.112*	
C40	0.5009 (5)	0.2480 (4)	0.6365 (5)	0.083 (2)	
H40A	0.4661	0.2103	0.6196	0.099*	
C41	1.0587 (5)	0.6350 (4)	0.6450 (5)	0.086 (2)	
H41A	1.1123	0.6430	0.6373	0.104*	
C42	0.7775 (5)	0.5773 (3)	0.8950 (4)	0.0636 (18)	
C43	0.7891 (5)	0.3628 (4)	0.8530 (4)	0.083 (2)	
H43A	0.8171	0.3272	0.8883	0.124*	
H43B	0.7239	0.3547	0.8242	0.124*	
H43C	0.7978	0.4020	0.8832	0.124*	
C44	0.9681 (5)	0.5663 (3)	0.9334 (4)	0.0710 (19)	
H44A	1.0313	0.5628	0.9465	0.085*	
C45	0.5130 (6)	0.5643 (4)	0.8099 (6)	0.092 (3)	
H45A	0.4799	0.5833	0.7571	0.110*	
C46	0.9355 (6)	0.6026 (3)	0.9818 (4)	0.078 (2)	
H46A	0.9777	0.6236	1.0266	0.093*	
C47	1.0930 (5)	0.3472 (4)	0.5980 (5)	0.082 (2)	
H47A	1.0493	0.3471	0.5420	0.098*	
C48	1.1323 (5)	0.4146 (4)	0.6139 (5)	0.080 (2)	
H48A	1.1814	0.4141	0.6678	0.096*	
C49	0.6957 (5)	0.3883 (4)	0.4187 (4)	0.086 (2)	
H49A	0.6352	0.3957	0.4176	0.130*	
H49B	0.7025	0.3432	0.4095	0.130*	
H49C	0.7027	0.4137	0.3781	0.130*	
C50	0.9808 (6)	0.2245 (4)	0.8471 (6)	0.104 (3)	
H50A	0.9973	0.1887	0.8805	0.125*	
C51	0.6292 (4)	0.3143 (3)	0.6436 (4)	0.0580 (16)	
C52	0.9369 (5)	0.6744 (4)	0.6782 (5)	0.085 (2)	
H52A	0.9084	0.7088	0.6918	0.102*	
C53	0.9153 (5)	0.2685 (3)	0.8499 (5)	0.081 (2)	
H53A	0.8876	0.2621	0.8849	0.097*	
C54	0.7392 (5)	0.2789 (3)	0.5959 (5)	0.084 (2)	
H54A	0.7950	0.2935	0.5924	0.126*	
H54B	0.6924	0.2708	0.5436	0.126*	
H54C	0.7517	0.2397	0.6265	0.126*	
C55	1.2101 (6)	0.3149 (5)	0.5481 (6)	0.116 (3)	
H55A	1.2603	0.2847	0.5565	0.139*	
H55B	1.1648	0.3102	0.4937	0.139*	
C56	1.2477 (6)	0.3828 (5)	0.5607 (7)	0.113 (3)	
H56A	1.3013	0.3849	0.6112	0.136*	
H56B	1.2685	0.3931	0.5195	0.136*	
C57	1.0206 (5)	0.2342 (4)	0.7947 (5)	0.090 (3)	
H57A	1.0636	0.2042	0.7932	0.109*	
C58	1.1641 (5)	0.2978 (4)	0.6045 (5)	0.093 (3)	
H58A	1.1351	0.2555	0.5910	0.112*	
H58B	1.2108	0.2962	0.6587	0.112*	
C59	0.5801 (5)	0.2566 (3)	0.6215 (4)	0.073 (2)	
H59A	0.5996	0.2240	0.5972	0.088*	
C60	1.0131 (6)	0.6856 (3)	0.6612 (5)	0.091 (3)	
H60A	1.0335	0.7278	0.6607	0.109*	
C64	0.3833 (7)	0.6040 (5)	0.8373 (7)	0.132 (4)	
H64A	0.3552	0.6268	0.7871	0.159*	
H64B	0.3672	0.6272	0.8755	0.159*	
C68	0.3431 (8)	0.5361 (5)	0.8271 (8)	0.143 (5)	
H68A	0.3623	0.5160	0.8788	0.172*	
H68B	0.2759	0.5388	0.8035	0.172*	
C69	0.3723 (6)	0.4957 (5)	0.7776 (6)	0.112 (3)	
H69A	0.3441	0.5117	0.7236	0.135*	
H69B	0.3498	0.4520	0.7776	0.135*	
C71	0.4879 (6)	0.6046 (5)	0.8652 (6)	0.110 (3)	
H71A	0.5095	0.6487	0.8660	0.132*	
H71B	0.5173	0.5872	0.9186	0.132*	
C72	0.2937 (6)	0.1233 (5)	0.5715 (7)	0.134 (4)	
H72A	0.2939	0.1166	0.6229	0.200*	
H72B	0.2667	0.1647	0.5512	0.200*	
H72C	0.3563	0.1221	0.5758	0.200*	
F1	0.2731 (10)	0.3061 (4)	0.9481 (7)	0.301 (7)	
F2	0.3984 (7)	0.2862 (6)	0.9409 (6)	0.267 (5)	
F3	0.2290 (6)	0.3579 (4)	0.8329 (7)	0.266 (6)	
F4	0.3428 (7)	0.3875 (4)	0.9315 (5)	0.233 (4)	
F5	0.3559 (9)	0.3351 (5)	0.8342 (7)	0.267 (6)	
F6	0.2767 (6)	0.2580 (4)	0.8468 (5)	0.202 (3)	
Gd1	0.78621 (2)	0.458889 (15)	0.683056 (19)	0.05067 (11)	
N1	0.6122 (4)	0.5603 (3)	0.8230 (4)	0.0769 (17)	
N2	0.5107 (4)	0.4567 (3)	0.7546 (4)	0.0702 (16)	
N3	1.0625 (3)	0.4607 (2)	0.6149 (3)	0.0592 (13)	
N4	1.0354 (4)	0.3387 (3)	0.6428 (4)	0.0648 (15)	
O1	0.7533 (3)	0.5113 (2)	0.7804 (3)	0.0581 (11)	
O2	0.6459 (3)	0.42001 (19)	0.6928 (3)	0.0596 (11)	
O3	0.9274 (3)	0.4990 (2)	0.8165 (3)	0.0650 (12)	
O4	0.7073 (3)	0.3274 (2)	0.6334 (3)	0.0687 (12)	
O5	0.9064 (3)	0.50117 (18)	0.6495 (2)	0.0510 (10)	
O6	0.9029 (3)	0.38335 (19)	0.6990 (2)	0.0566 (10)	
O7	0.8278 (3)	0.59487 (19)	0.6900 (3)	0.0623 (11)	
O8	0.8311 (3)	0.3694 (2)	0.7990 (3)	0.0659 (12)	
O9	0.5511 (3)	0.5546 (2)	0.6358 (3)	0.0659 (12)	
O10	0.6651 (3)	0.5276 (2)	0.6023 (3)	0.0599 (11)	
O11	0.7443 (3)	0.4268 (2)	0.5507 (3)	0.0613 (11)	
O12	0.8534 (3)	0.4006 (2)	0.5098 (3)	0.0630 (12)	
O13	0.2345 (9)	0.0677 (6)	0.5128 (7)	0.257 (5)	
H131	0.1778	0.0631	0.4813	0.386*	
O14	0.1290 (6)	0.1453 (5)	0.4517 (6)	0.217 (4)	
H142	0.1215	0.1713	0.4139	0.325*	
H141	0.0880	0.1261	0.4623	0.325*	
P1	0.3108 (2)	0.32265 (15)	0.88998 (18)	0.1102 (8)	
Zn2	0.94810 (4)	0.41679 (3)	0.61748 (4)	0.05187 (19)	
Zn3	0.61201 (5)	0.50326 (4)	0.73265 (5)	0.0604 (2)	

### Atomic displacement parameters (Å^2^)

**Table:**

	*U*^11^	*U*^22^	*U*^33^	*U*^12^	*U*^13^	*U*^23^
C15	0.072 (4)	0.045 (3)	0.045 (4)	0.001 (3)	0.024 (3)	0.000 (3)
C17	0.061 (4)	0.052 (4)	0.062 (4)	−0.007 (3)	0.029 (3)	0.000 (3)
C19	0.059 (4)	0.058 (4)	0.066 (5)	−0.003 (3)	0.022 (4)	0.010 (3)
C20	0.073 (4)	0.048 (3)	0.049 (4)	0.001 (3)	0.020 (4)	0.006 (3)
C21	0.059 (4)	0.047 (3)	0.057 (4)	−0.004 (3)	0.019 (3)	0.004 (3)
C22	0.101 (6)	0.062 (4)	0.076 (5)	−0.004 (4)	0.061 (5)	−0.009 (4)
C23	0.060 (4)	0.068 (5)	0.081 (5)	−0.014 (3)	0.034 (4)	0.001 (4)
C24	0.065 (4)	0.046 (3)	0.061 (4)	−0.005 (3)	0.028 (3)	0.001 (3)
C25	0.056 (3)	0.047 (3)	0.044 (4)	−0.011 (3)	0.014 (3)	0.004 (3)
C26	0.061 (4)	0.070 (5)	0.087 (5)	−0.009 (4)	0.040 (4)	0.009 (4)
C27	0.105 (6)	0.070 (5)	0.133 (8)	−0.005 (4)	0.083 (6)	−0.009 (5)
C28	0.045 (3)	0.057 (4)	0.052 (4)	−0.005 (3)	0.013 (3)	0.003 (3)
C29	0.067 (4)	0.049 (4)	0.087 (5)	0.012 (3)	0.032 (4)	0.014 (4)
C30	0.054 (4)	0.047 (4)	0.065 (4)	−0.004 (3)	0.023 (3)	−0.004 (3)
C31	0.065 (4)	0.062 (4)	0.101 (6)	−0.008 (4)	0.035 (4)	0.010 (4)
C32	0.059 (4)	0.063 (4)	0.117 (7)	0.016 (3)	0.044 (5)	0.016 (5)
C33	0.063 (4)	0.106 (6)	0.063 (5)	0.002 (4)	0.019 (4)	0.005 (5)
C34	0.060 (4)	0.050 (4)	0.060 (4)	−0.001 (3)	0.019 (3)	0.010 (3)
C35	0.056 (4)	0.097 (5)	0.094 (6)	0.005 (4)	0.041 (4)	0.013 (5)
C36	0.055 (4)	0.048 (3)	0.056 (4)	−0.010 (3)	0.017 (3)	−0.004 (3)
C37	0.081 (5)	0.065 (4)	0.094 (6)	0.001 (4)	0.044 (5)	−0.009 (4)
C38	0.112 (6)	0.061 (4)	0.056 (5)	0.000 (4)	0.038 (5)	−0.001 (4)
C39	0.069 (4)	0.079 (5)	0.077 (5)	0.013 (4)	0.031 (4)	0.011 (4)
C40	0.073 (5)	0.059 (4)	0.108 (7)	−0.019 (4)	0.029 (5)	0.000 (5)
C41	0.092 (5)	0.078 (5)	0.108 (7)	−0.030 (4)	0.060 (5)	−0.014 (5)
C42	0.092 (5)	0.044 (4)	0.052 (4)	−0.004 (3)	0.027 (4)	−0.003 (3)
C43	0.088 (5)	0.095 (6)	0.076 (5)	0.006 (4)	0.046 (5)	0.016 (5)
C44	0.069 (4)	0.063 (4)	0.067 (5)	−0.001 (4)	0.014 (4)	0.007 (4)
C45	0.087 (5)	0.102 (6)	0.120 (7)	−0.010 (5)	0.075 (6)	−0.022 (6)
C46	0.095 (6)	0.064 (5)	0.056 (5)	−0.014 (4)	0.013 (4)	−0.007 (4)
C47	0.078 (5)	0.071 (5)	0.117 (7)	0.010 (4)	0.061 (5)	0.003 (5)
C48	0.076 (5)	0.078 (5)	0.108 (6)	0.009 (4)	0.061 (5)	0.006 (5)
C49	0.064 (4)	0.127 (7)	0.060 (5)	−0.018 (4)	0.016 (4)	−0.023 (5)
C50	0.101 (6)	0.084 (6)	0.140 (8)	0.034 (5)	0.061 (6)	0.068 (6)
C51	0.059 (4)	0.052 (4)	0.060 (4)	0.000 (3)	0.021 (3)	−0.003 (3)
C52	0.096 (6)	0.057 (4)	0.106 (7)	−0.011 (4)	0.047 (5)	−0.018 (4)
C53	0.084 (5)	0.066 (5)	0.094 (6)	−0.011 (4)	0.039 (5)	0.016 (4)
C54	0.091 (5)	0.061 (4)	0.110 (7)	−0.004 (4)	0.051 (5)	−0.023 (4)
C55	0.089 (6)	0.142 (9)	0.137 (9)	0.024 (6)	0.066 (6)	−0.006 (7)
C56	0.100 (6)	0.117 (8)	0.150 (9)	0.018 (6)	0.079 (7)	0.015 (7)
C57	0.089 (5)	0.068 (5)	0.125 (7)	0.029 (4)	0.054 (6)	0.040 (5)
C58	0.083 (5)	0.086 (5)	0.130 (8)	0.016 (4)	0.064 (6)	0.010 (5)
C59	0.068 (4)	0.058 (4)	0.084 (5)	−0.010 (3)	0.023 (4)	0.000 (4)
C60	0.108 (6)	0.052 (4)	0.140 (8)	−0.033 (4)	0.079 (6)	−0.023 (5)
C64	0.104 (7)	0.129 (9)	0.182 (12)	0.006 (6)	0.076 (8)	−0.032 (8)
C68	0.132 (9)	0.125 (9)	0.213 (14)	−0.020 (7)	0.113 (10)	−0.027 (9)
C69	0.108 (7)	0.115 (7)	0.145 (9)	−0.029 (6)	0.084 (7)	−0.041 (7)
C71	0.111 (7)	0.113 (7)	0.117 (8)	0.004 (6)	0.057 (6)	−0.022 (6)
C72	0.083 (5)	0.135 (7)	0.198 (9)	−0.013 (5)	0.073 (6)	0.013 (7)
F1	0.56 (2)	0.176 (8)	0.393 (15)	0.020 (10)	0.424 (16)	0.004 (8)
F2	0.237 (10)	0.314 (13)	0.179 (8)	0.091 (10)	0.013 (8)	0.008 (9)
F3	0.206 (8)	0.193 (9)	0.272 (12)	0.078 (7)	−0.030 (8)	−0.040 (8)
F4	0.324 (11)	0.155 (7)	0.159 (7)	−0.036 (7)	0.039 (7)	−0.061 (6)
F5	0.399 (15)	0.215 (9)	0.345 (14)	−0.019 (10)	0.311 (13)	0.025 (9)
F6	0.254 (9)	0.143 (6)	0.236 (9)	−0.032 (6)	0.127 (8)	−0.059 (6)
Gd1	0.05211 (18)	0.05026 (18)	0.0530 (2)	0.00167 (14)	0.02512 (15)	−0.00155 (16)
N1	0.087 (4)	0.070 (4)	0.089 (5)	−0.010 (3)	0.051 (4)	−0.020 (4)
N2	0.074 (4)	0.065 (4)	0.087 (4)	−0.001 (3)	0.049 (3)	−0.004 (3)
N3	0.059 (3)	0.056 (3)	0.071 (4)	−0.001 (3)	0.035 (3)	0.003 (3)
N4	0.061 (3)	0.055 (3)	0.088 (4)	0.010 (3)	0.041 (3)	0.015 (3)
O1	0.058 (2)	0.064 (3)	0.055 (3)	−0.011 (2)	0.026 (2)	−0.014 (2)
O2	0.066 (3)	0.049 (2)	0.072 (3)	−0.009 (2)	0.036 (2)	−0.007 (2)
O3	0.062 (3)	0.071 (3)	0.061 (3)	−0.001 (2)	0.024 (2)	−0.001 (3)
O4	0.075 (3)	0.054 (3)	0.087 (4)	−0.005 (2)	0.043 (3)	−0.010 (3)
O5	0.055 (2)	0.044 (2)	0.058 (3)	−0.0039 (18)	0.028 (2)	0.002 (2)
O6	0.061 (2)	0.051 (2)	0.064 (3)	0.006 (2)	0.032 (2)	0.009 (2)
O7	0.066 (3)	0.049 (2)	0.080 (3)	0.004 (2)	0.037 (2)	0.000 (2)
O8	0.082 (3)	0.063 (3)	0.066 (3)	0.004 (2)	0.043 (3)	0.010 (2)
O9	0.069 (3)	0.062 (3)	0.075 (3)	0.009 (2)	0.037 (3)	0.002 (2)
O10	0.056 (2)	0.057 (3)	0.066 (3)	0.006 (2)	0.025 (2)	0.002 (2)
O11	0.059 (3)	0.067 (3)	0.055 (3)	−0.001 (2)	0.022 (2)	−0.010 (2)
O12	0.055 (3)	0.072 (3)	0.061 (3)	−0.004 (2)	0.023 (2)	−0.015 (2)
O13	0.309 (9)	0.271 (9)	0.243 (9)	−0.026 (8)	0.165 (8)	0.022 (7)
O14	0.206 (7)	0.276 (9)	0.183 (7)	0.028 (7)	0.095 (6)	−0.033 (6)
P1	0.135 (2)	0.115 (2)	0.098 (2)	0.0004 (19)	0.065 (2)	−0.0109 (18)
Zn2	0.0487 (4)	0.0502 (4)	0.0584 (5)	0.0007 (3)	0.0239 (4)	0.0012 (3)
Zn3	0.0629 (5)	0.0566 (4)	0.0713 (5)	−0.0020 (4)	0.0374 (4)	−0.0042 (4)

### Geometric parameters (Å, º)

**Table:**

C15—O1	1.328 (7)	C47—N4	1.473 (8)
C15—C20	1.403 (9)	C47—C58	1.485 (9)
C15—C42	1.411 (9)	C47—C48	1.502 (10)
C17—C25	1.394 (8)	C47—H47A	0.9800
C17—C41	1.424 (9)	C48—N3	1.464 (8)
C17—C23	1.428 (9)	C48—H48A	0.9800
C19—C28	1.410 (8)	C49—H49A	0.9600
C19—C31	1.412 (9)	C49—H49B	0.9600
C19—C26	1.443 (9)	C49—H49C	0.9600
C20—C44	1.358 (9)	C50—C57	1.370 (11)
C20—O3	1.379 (7)	C50—C53	1.394 (10)
C21—O6	1.326 (7)	C50—H50A	0.9300
C21—C34	1.404 (9)	C51—O4	1.351 (7)
C21—C29	1.425 (9)	C51—C59	1.388 (8)
C22—N1	1.282 (9)	C52—C60	1.384 (10)
C22—C42	1.444 (10)	C52—H52A	0.9300
C22—H22A	0.9300	C53—H53A	0.9300
C23—N3	1.265 (8)	C54—O4	1.422 (7)
C23—H23A	0.9300	C54—H54A	0.9600
C24—O7	1.371 (7)	C54—H54B	0.9600
C24—C52	1.393 (9)	C54—H54C	0.9600
C24—C25	1.426 (8)	C55—C56	1.503 (12)
C25—O5	1.333 (6)	C55—C58	1.539 (11)
C26—N2	1.277 (8)	C55—H55A	0.9700
C26—H26A	0.9300	C55—H55B	0.9700
C27—N2	1.457 (9)	C56—H56A	0.9700
C27—C69	1.517 (11)	C56—H56B	0.9700
C27—C45	1.558 (11)	C57—H57A	0.9300
C27—H27A	0.9800	C58—H58A	0.9700
C28—O2	1.326 (7)	C58—H58B	0.9700
C28—C51	1.408 (8)	C59—H59A	0.9300
C29—C57	1.393 (9)	C60—H60A	0.9300
C29—C32	1.450 (10)	C64—C71	1.517 (12)
C30—O9	1.248 (7)	C64—C68	1.518 (13)
C30—O10	1.254 (7)	C64—H64A	0.9700
C30—C39	1.501 (9)	C64—H64B	0.9700
C31—C40	1.345 (10)	C68—C69	1.448 (12)
C31—H31A	0.9300	C68—H68A	0.9700
C32—N4	1.255 (8)	C68—H68B	0.9700
C32—H32A	0.9300	C69—H69A	0.9700
C33—O3	1.434 (7)	C69—H69B	0.9700
C33—H33A	0.9600	C71—H71A	0.9700
C33—H33B	0.9600	C71—H71B	0.9700
C33—H33C	0.9600	C72—O13	1.592 (8)
C34—C53	1.373 (9)	C72—H72A	0.9600
C34—O8	1.379 (7)	C72—H72B	0.9600
C35—C56	1.500 (10)	C72—H72C	0.9600
C35—C48	1.512 (10)	F1—P1	1.469 (8)
C35—H35A	0.9700	F2—P1	1.515 (9)
C35—H35B	0.9700	F3—P1	1.480 (8)
C36—O11	1.234 (7)	F4—P1	1.522 (8)
C36—O12	1.267 (7)	F5—P1	1.499 (8)
C36—C49	1.524 (8)	F6—P1	1.534 (7)
C37—O7	1.422 (7)	Gd1—O1	2.337 (4)
C37—H37A	0.9600	Gd1—O6	2.340 (4)
C37—H37B	0.9600	Gd1—O11	2.353 (4)
C37—H37C	0.9600	Gd1—O10	2.356 (4)
C38—C46	1.341 (10)	Gd1—O5	2.394 (4)
C38—C42	1.409 (9)	Gd1—O2	2.435 (4)
C38—H38A	0.9300	Gd1—O3	2.686 (4)
C39—H39A	0.9600	Gd1—O8	2.696 (4)
C39—H39B	0.9600	Gd1—O7	2.874 (4)
C39—H39C	0.9600	Gd1—Zn2	3.3679 (8)
C40—C59	1.401 (10)	Gd1—Zn3	3.3697 (8)
C40—H40A	0.9300	N1—Zn3	2.044 (6)
C41—C60	1.369 (10)	N2—Zn3	2.046 (6)
C41—H41A	0.9300	N3—Zn2	2.041 (5)
C43—O8	1.415 (8)	N4—Zn2	2.047 (5)
C43—H43A	0.9600	O1—Zn3	2.042 (4)
C43—H43B	0.9600	O2—Zn3	2.027 (4)
C43—H43C	0.9600	O5—Zn2	2.035 (4)
C44—C46	1.417 (10)	O6—Zn2	2.038 (4)
C44—H44A	0.9300	O9—Zn3	1.960 (5)
C45—N1	1.488 (9)	O12—Zn2	1.965 (4)
C45—C71	1.492 (11)	O13—H131	0.8500
C45—H45A	0.9800	O14—H142	0.8500
C46—H46A	0.9300	O14—H141	0.8500
			
O1—C15—C20	117.5 (6)	C41—C60—H60A	119.7
O1—C15—C42	123.5 (6)	C52—C60—H60A	119.7
C20—C15—C42	119.0 (6)	C71—C64—C68	113.0 (9)
C25—C17—C41	119.2 (6)	C71—C64—H64A	109.0
C25—C17—C23	124.0 (6)	C68—C64—H64A	109.0
C41—C17—C23	116.7 (6)	C71—C64—H64B	109.0
C28—C19—C31	119.4 (7)	C68—C64—H64B	109.0
C28—C19—C26	125.1 (6)	H64A—C64—H64B	107.8
C31—C19—C26	115.5 (6)	C69—C68—C64	112.6 (9)
C44—C20—O3	124.7 (6)	C69—C68—H68A	109.1
C44—C20—C15	121.9 (7)	C64—C68—H68A	109.1
O3—C20—C15	113.4 (6)	C69—C68—H68B	109.1
O6—C21—C34	117.5 (6)	C64—C68—H68B	109.1
O6—C21—C29	123.9 (6)	H68A—C68—H68B	107.8
C34—C21—C29	118.6 (6)	C68—C69—C27	113.6 (9)
N1—C22—C42	125.0 (7)	C68—C69—H69A	108.9
N1—C22—H22A	117.5	C27—C69—H69A	108.9
C42—C22—H22A	117.5	C68—C69—H69B	108.9
N3—C23—C17	129.2 (6)	C27—C69—H69B	108.9
N3—C23—H23A	115.4	H69A—C69—H69B	107.7
C17—C23—H23A	115.4	C45—C71—C64	108.9 (8)
O7—C24—C52	125.3 (6)	C45—C71—H71A	109.9
O7—C24—C25	114.6 (5)	C64—C71—H71A	109.9
C52—C24—C25	120.1 (6)	C45—C71—H71B	109.9
O5—C25—C17	125.4 (6)	C64—C71—H71B	109.9
O5—C25—C24	115.5 (5)	H71A—C71—H71B	108.3
C17—C25—C24	119.0 (5)	O13—C72—H72A	109.5
N2—C26—C19	126.4 (6)	O13—C72—H72B	109.5
N2—C26—H26A	116.8	H72A—C72—H72B	109.5
C19—C26—H26A	116.8	O13—C72—H72C	109.5
N2—C27—C69	116.4 (8)	H72A—C72—H72C	109.5
N2—C27—C45	108.1 (6)	H72B—C72—H72C	109.5
C69—C27—C45	108.3 (7)	O1—Gd1—O6	128.77 (15)
N2—C27—H27A	107.9	O1—Gd1—O11	151.18 (15)
C69—C27—H27A	107.9	O6—Gd1—O11	79.19 (15)
C45—C27—H27A	107.9	O1—Gd1—O10	80.13 (15)
O2—C28—C51	117.3 (5)	O6—Gd1—O10	150.90 (15)
O2—C28—C19	124.4 (6)	O11—Gd1—O10	72.75 (15)
C51—C28—C19	118.2 (6)	O1—Gd1—O5	118.96 (14)
C57—C29—C21	117.7 (7)	O6—Gd1—O5	66.83 (13)
C57—C29—C32	118.6 (6)	O11—Gd1—O5	75.26 (14)
C21—C29—C32	123.7 (6)	O10—Gd1—O5	98.10 (14)
O9—C30—O10	123.6 (6)	O1—Gd1—O2	66.18 (14)
O9—C30—C39	117.3 (6)	O6—Gd1—O2	117.80 (13)
O10—C30—C39	119.1 (6)	O11—Gd1—O2	96.33 (14)
C40—C31—C19	120.9 (7)	O10—Gd1—O2	73.61 (14)
C40—C31—H31A	119.5	O5—Gd1—O2	169.83 (14)
C19—C31—H31A	119.5	O1—Gd1—O3	60.92 (14)
N4—C32—C29	126.2 (6)	O6—Gd1—O3	77.05 (14)
N4—C32—H32A	116.9	O11—Gd1—O3	144.44 (14)
C29—C32—H32A	116.9	O10—Gd1—O3	123.19 (14)
O3—C33—H33A	109.5	O5—Gd1—O3	71.23 (13)
O3—C33—H33B	109.5	O2—Gd1—O3	118.10 (14)
H33A—C33—H33B	109.5	O1—Gd1—O8	76.86 (14)
O3—C33—H33C	109.5	O6—Gd1—O8	60.53 (13)
H33A—C33—H33C	109.5	O11—Gd1—O8	120.28 (14)
H33B—C33—H33C	109.5	O10—Gd1—O8	142.69 (14)
C53—C34—O8	125.1 (7)	O5—Gd1—O8	118.69 (13)
C53—C34—C21	121.9 (6)	O2—Gd1—O8	70.35 (14)
O8—C34—C21	113.0 (5)	O3—Gd1—O8	68.51 (14)
C56—C35—C48	111.6 (7)	O1—Gd1—O7	68.47 (14)
C56—C35—H35A	109.3	O6—Gd1—O7	119.41 (13)
C48—C35—H35A	109.3	O11—Gd1—O7	106.41 (14)
C56—C35—H35B	109.3	O10—Gd1—O7	63.37 (13)
C48—C35—H35B	109.3	O5—Gd1—O7	57.58 (12)
H35A—C35—H35B	108.0	O2—Gd1—O7	121.16 (13)
O11—C36—O12	124.8 (6)	O3—Gd1—O7	64.52 (13)
O11—C36—C49	118.5 (6)	O8—Gd1—O7	130.82 (13)
O12—C36—C49	116.7 (6)	O1—Gd1—Zn2	147.29 (10)
O7—C37—H37A	109.5	O6—Gd1—Zn2	36.53 (10)
O7—C37—H37B	109.5	O11—Gd1—Zn2	58.79 (10)
H37A—C37—H37B	109.5	O10—Gd1—Zn2	117.82 (11)
O7—C37—H37C	109.5	O5—Gd1—Zn2	36.68 (9)
H37A—C37—H37C	109.5	O2—Gd1—Zn2	142.45 (10)
H37B—C37—H37C	109.5	O3—Gd1—Zn2	86.76 (10)
C46—C38—C42	122.0 (7)	O8—Gd1—Zn2	96.93 (9)
C46—C38—H38A	119.0	O7—Gd1—Zn2	94.24 (9)
C42—C38—H38A	119.0	O1—Gd1—Zn3	36.59 (10)
C30—C39—H39A	109.5	O6—Gd1—Zn3	146.88 (10)
C30—C39—H39B	109.5	O11—Gd1—Zn3	116.70 (10)
H39A—C39—H39B	109.5	O10—Gd1—Zn3	57.79 (11)
C30—C39—H39C	109.5	O5—Gd1—Zn3	142.85 (9)
H39A—C39—H39C	109.5	O2—Gd1—Zn3	36.61 (9)
H39B—C39—H39C	109.5	O3—Gd1—Zn3	97.40 (10)
C31—C40—C59	120.9 (7)	O8—Gd1—Zn3	86.88 (9)
C31—C40—H40A	119.5	O7—Gd1—Zn3	85.43 (9)
C59—C40—H40A	119.5	Zn2—Gd1—Zn3	175.19 (2)
C60—C41—C17	120.7 (7)	C22—N1—C45	126.0 (7)
C60—C41—H41A	119.6	C22—N1—Zn3	129.0 (5)
C17—C41—H41A	119.6	C45—N1—Zn3	104.7 (5)
C38—C42—C15	117.9 (7)	C26—N2—C27	120.0 (6)
C38—C42—C22	117.1 (7)	C26—N2—Zn3	125.2 (5)
C15—C42—C22	125.0 (6)	C27—N2—Zn3	113.8 (5)
O8—C43—H43A	109.5	C23—N3—C48	121.9 (6)
O8—C43—H43B	109.5	C23—N3—Zn2	124.1 (5)
H43A—C43—H43B	109.5	C48—N3—Zn2	113.1 (4)
O8—C43—H43C	109.5	C32—N4—C47	123.6 (6)
H43A—C43—H43C	109.5	C32—N4—Zn2	128.7 (5)
H43B—C43—H43C	109.5	C47—N4—Zn2	107.0 (4)
C20—C44—C46	118.8 (7)	C15—O1—Zn3	129.2 (4)
C20—C44—H44A	120.6	C15—O1—Gd1	130.3 (4)
C46—C44—H44A	120.6	Zn3—O1—Gd1	100.39 (17)
N1—C45—C71	118.7 (7)	C28—O2—Zn3	126.2 (4)
N1—C45—C27	107.0 (6)	C28—O2—Gd1	136.1 (4)
C71—C45—C27	111.9 (7)	Zn3—O2—Gd1	97.65 (15)
N1—C45—H45A	106.1	C20—O3—C33	117.7 (5)
C71—C45—H45A	106.1	C20—O3—Gd1	117.0 (4)
C27—C45—H45A	106.1	C33—O3—Gd1	124.8 (4)
C38—C46—C44	120.5 (7)	C51—O4—C54	117.2 (5)
C38—C46—H46A	119.8	C25—O5—Zn2	125.4 (4)
C44—C46—H46A	119.8	C25—O5—Gd1	135.8 (4)
N4—C47—C58	119.7 (7)	Zn2—O5—Gd1	98.68 (15)
N4—C47—C48	107.6 (6)	C21—O6—Zn2	128.7 (4)
C58—C47—C48	112.5 (6)	C21—O6—Gd1	130.9 (4)
N4—C47—H47A	105.3	Zn2—O6—Gd1	100.36 (16)
C58—C47—H47A	105.3	C24—O7—C37	115.9 (5)
C48—C47—H47A	105.3	C24—O7—Gd1	116.1 (3)
N3—C48—C47	110.5 (6)	C37—O7—Gd1	127.6 (4)
N3—C48—C35	114.9 (6)	C34—O8—C43	116.6 (5)
C47—C48—C35	112.2 (7)	C34—O8—Gd1	117.4 (4)
N3—C48—H48A	106.2	C43—O8—Gd1	125.6 (4)
C47—C48—H48A	106.2	C30—O9—Zn3	116.3 (4)
C35—C48—H48A	106.2	C30—O10—Gd1	148.9 (4)
C36—C49—H49A	109.5	C36—O11—Gd1	147.9 (4)
C36—C49—H49B	109.5	C36—O12—Zn2	116.5 (4)
H49A—C49—H49B	109.5	C72—O13—H131	134.9
C36—C49—H49C	109.5	H142—O14—H141	128.6
H49A—C49—H49C	109.5	F1—P1—F3	97.9 (7)
H49B—C49—H49C	109.5	F1—P1—F5	174.9 (7)
C57—C50—C53	119.7 (7)	F3—P1—F5	87.1 (7)
C57—C50—H50A	120.2	F1—P1—F2	88.4 (7)
C53—C50—H50A	120.2	F3—P1—F2	173.5 (7)
O4—C51—C59	124.2 (6)	F5—P1—F2	86.6 (6)
O4—C51—C28	114.8 (5)	F1—P1—F4	89.2 (6)
C59—C51—C28	121.0 (6)	F3—P1—F4	86.4 (5)
C60—C52—C24	120.2 (7)	F5—P1—F4	92.4 (6)
C60—C52—H52A	119.9	F2—P1—F4	95.4 (6)
C24—C52—H52A	119.9	F1—P1—F6	91.0 (6)
C34—C53—C50	119.3 (7)	F3—P1—F6	92.3 (5)
C34—C53—H53A	120.3	F5—P1—F6	87.5 (5)
C50—C53—H53A	120.3	F2—P1—F6	85.8 (6)
O4—C54—H54A	109.5	F4—P1—F6	178.8 (6)
O4—C54—H54B	109.5	O12—Zn2—O5	103.75 (17)
H54A—C54—H54B	109.5	O12—Zn2—O6	109.85 (18)
O4—C54—H54C	109.5	O5—Zn2—O6	79.60 (16)
H54A—C54—H54C	109.5	O12—Zn2—N3	111.4 (2)
H54B—C54—H54C	109.5	O5—Zn2—N3	91.34 (18)
C56—C55—C58	112.1 (8)	O6—Zn2—N3	138.8 (2)
C56—C55—H55A	109.2	O12—Zn2—N4	105.0 (2)
C58—C55—H55A	109.2	O5—Zn2—N4	151.2 (2)
C56—C55—H55B	109.2	O6—Zn2—N4	88.47 (19)
C58—C55—H55B	109.2	N3—Zn2—N4	80.5 (2)
H55A—C55—H55B	107.9	O12—Zn2—Gd1	91.96 (13)
C35—C56—C55	113.8 (7)	O5—Zn2—Gd1	44.64 (10)
C35—C56—H56A	108.8	O6—Zn2—Gd1	43.11 (11)
C55—C56—H56A	108.8	N3—Zn2—Gd1	134.92 (15)
C35—C56—H56B	108.8	N4—Zn2—Gd1	131.45 (16)
C55—C56—H56B	108.8	O9—Zn3—O2	103.26 (18)
H56A—C56—H56B	107.7	O9—Zn3—O1	112.88 (18)
C50—C57—C29	122.7 (7)	O2—Zn3—O1	79.67 (17)
C50—C57—H57A	118.6	O9—Zn3—N1	105.7 (2)
C29—C57—H57A	118.6	O2—Zn3—N1	151.0 (2)
C47—C58—C55	110.0 (7)	O1—Zn3—N1	88.1 (2)
C47—C58—H58A	109.7	O9—Zn3—N2	107.8 (2)
C55—C58—H58A	109.7	O2—Zn3—N2	91.1 (2)
C47—C58—H58B	109.7	O1—Zn3—N2	139.3 (2)
C55—C58—H58B	109.7	N1—Zn3—N2	81.1 (2)
H58A—C58—H58B	108.2	O9—Zn3—Gd1	92.65 (13)
C51—C59—C40	119.2 (7)	O2—Zn3—Gd1	45.74 (11)
C51—C59—H59A	120.4	O1—Zn3—Gd1	43.02 (12)
C40—C59—H59A	120.4	N1—Zn3—Gd1	130.82 (18)
C41—C60—C52	120.5 (7)	N2—Zn3—Gd1	136.11 (16)
			
O1—C15—C20—C44	179.7 (6)	O3—Gd1—O7—C24	78.4 (4)
C42—C15—C20—C44	−1.3 (9)	O8—Gd1—O7—C24	97.0 (4)
O1—C15—C20—O3	−1.1 (8)	Zn2—Gd1—O7—C24	−6.0 (4)
C42—C15—C20—O3	177.9 (5)	Zn3—Gd1—O7—C24	178.9 (4)
C25—C17—C23—N3	8.3 (12)	O1—Gd1—O7—C37	−42.7 (5)
C41—C17—C23—N3	−175.8 (8)	O6—Gd1—O7—C37	−166.1 (5)
C41—C17—C25—O5	178.9 (6)	O11—Gd1—O7—C37	107.3 (5)
C23—C17—C25—O5	−5.2 (10)	O10—Gd1—O7—C37	46.8 (5)
C41—C17—C25—C24	−2.3 (10)	O5—Gd1—O7—C37	167.2 (6)
C23—C17—C25—C24	173.5 (6)	O2—Gd1—O7—C37	−0.9 (6)
O7—C24—C25—O5	1.4 (8)	O3—Gd1—O7—C37	−109.7 (5)
C52—C24—C25—O5	−177.8 (6)	O8—Gd1—O7—C37	−91.1 (5)
O7—C24—C25—C17	−177.5 (5)	Zn2—Gd1—O7—C37	165.9 (5)
C52—C24—C25—C17	3.3 (10)	Zn3—Gd1—O7—C37	−9.2 (5)
C28—C19—C26—N2	13.6 (12)	C53—C34—O8—C43	−0.3 (9)
C31—C19—C26—N2	−169.6 (7)	C21—C34—O8—C43	−179.4 (6)
C31—C19—C28—O2	177.4 (6)	C53—C34—O8—Gd1	−173.0 (5)
C26—C19—C28—O2	−5.8 (10)	C21—C34—O8—Gd1	7.8 (7)
C31—C19—C28—C51	−3.6 (9)	O1—Gd1—O8—C34	−157.9 (4)
C26—C19—C28—C51	173.1 (6)	O6—Gd1—O8—C34	−7.4 (4)
O6—C21—C29—C57	−178.2 (6)	O11—Gd1—O8—C34	47.2 (4)
C34—C21—C29—C57	2.3 (10)	O10—Gd1—O8—C34	148.7 (4)
O6—C21—C29—C32	4.3 (11)	O5—Gd1—O8—C34	−41.9 (4)
C34—C21—C29—C32	−175.2 (7)	O2—Gd1—O8—C34	133.0 (4)
C28—C19—C31—C40	−1.4 (11)	O3—Gd1—O8—C34	−94.3 (4)
C26—C19—C31—C40	−178.4 (7)	O7—Gd1—O8—C34	−112.3 (4)
C57—C29—C32—N4	176.2 (8)	Zn2—Gd1—O8—C34	−10.6 (4)
C21—C29—C32—N4	−6.3 (13)	Zn3—Gd1—O8—C34	166.5 (4)
O6—C21—C34—C53	178.3 (6)	O1—Gd1—O8—C43	30.0 (5)
C29—C21—C34—C53	−2.1 (10)	O6—Gd1—O8—C43	−179.5 (6)
O6—C21—C34—O8	−2.6 (8)	O11—Gd1—O8—C43	−124.9 (5)
C29—C21—C34—O8	177.0 (6)	O10—Gd1—O8—C43	−23.4 (6)
C19—C31—C40—C59	4.8 (12)	O5—Gd1—O8—C43	146.1 (5)
C25—C17—C41—C60	−1.4 (12)	O2—Gd1—O8—C43	−39.0 (5)
C23—C17—C41—C60	−177.6 (8)	O3—Gd1—O8—C43	93.6 (5)
C46—C38—C42—C15	1.0 (10)	O7—Gd1—O8—C43	75.6 (5)
C46—C38—C42—C22	178.0 (7)	Zn2—Gd1—O8—C43	177.4 (5)
O1—C15—C42—C38	179.5 (6)	Zn3—Gd1—O8—C43	−5.6 (5)
C20—C15—C42—C38	0.5 (9)	O10—C30—O9—Zn3	−7.7 (8)
O1—C15—C42—C22	2.7 (10)	C39—C30—O9—Zn3	173.1 (4)
C20—C15—C42—C22	−176.2 (6)	O9—C30—O10—Gd1	13.5 (12)
N1—C22—C42—C38	178.8 (7)	C39—C30—O10—Gd1	−167.3 (5)
N1—C22—C42—C15	−4.4 (11)	O1—Gd1—O10—C30	−39.6 (8)
O3—C20—C44—C46	−178.6 (6)	O6—Gd1—O10—C30	146.4 (7)
C15—C20—C44—C46	0.6 (10)	O11—Gd1—O10—C30	130.5 (8)
N2—C27—C45—N1	−43.8 (9)	O5—Gd1—O10—C30	−157.8 (8)
C69—C27—C45—N1	−170.8 (7)	O2—Gd1—O10—C30	28.3 (8)
N2—C27—C45—C71	−175.5 (7)	O3—Gd1—O10—C30	−84.9 (8)
C69—C27—C45—C71	57.6 (11)	O8—Gd1—O10—C30	12.9 (9)
C42—C38—C46—C44	−1.8 (11)	O7—Gd1—O10—C30	−110.4 (8)
C20—C44—C46—C38	1.0 (11)	Zn2—Gd1—O10—C30	169.5 (7)
N4—C47—C48—N3	−40.2 (9)	Zn3—Gd1—O10—C30	−8.2 (7)
C58—C47—C48—N3	−174.1 (7)	O12—C36—O11—Gd1	−3.1 (12)
N4—C47—C48—C35	−169.9 (6)	C49—C36—O11—Gd1	173.7 (6)
C58—C47—C48—C35	56.2 (10)	O1—Gd1—O11—C36	160.9 (7)
C56—C35—C48—N3	−178.7 (7)	O6—Gd1—O11—C36	−31.8 (8)
C56—C35—C48—C47	−51.3 (10)	O10—Gd1—O11—C36	140.4 (8)
O2—C28—C51—O4	3.6 (8)	O5—Gd1—O11—C36	36.8 (8)
C19—C28—C51—O4	−175.4 (6)	O2—Gd1—O11—C36	−149.0 (8)
O2—C28—C51—C59	−175.7 (6)	O3—Gd1—O11—C36	16.9 (9)
C19—C28—C51—C59	5.3 (10)	O8—Gd1—O11—C36	−78.1 (8)
O7—C24—C52—C60	−179.6 (7)	O7—Gd1—O11—C36	85.9 (8)
C25—C24—C52—C60	−0.5 (12)	Zn2—Gd1—O11—C36	1.0 (7)
O8—C34—C53—C50	−177.8 (7)	Zn3—Gd1—O11—C36	179.0 (7)
C21—C34—C53—C50	1.2 (11)	O11—C36—O12—Zn2	3.3 (8)
C57—C50—C53—C34	−0.5 (13)	C49—C36—O12—Zn2	−173.5 (5)
C48—C35—C56—C55	49.7 (12)	C36—O12—Zn2—O5	−45.7 (5)
C58—C55—C56—C35	−51.2 (12)	C36—O12—Zn2—O6	38.0 (5)
C53—C50—C57—C29	0.7 (14)	C36—O12—Zn2—N3	−142.7 (4)
C21—C29—C57—C50	−1.6 (13)	C36—O12—Zn2—N4	131.9 (4)
C32—C29—C57—C50	176.0 (8)	C36—O12—Zn2—Gd1	−2.1 (4)
N4—C47—C58—C55	176.0 (7)	C25—O5—Zn2—O12	−105.7 (4)
C48—C47—C58—C55	−56.2 (11)	Gd1—O5—Zn2—O12	78.60 (18)
C56—C55—C58—C47	53.5 (11)	C25—O5—Zn2—O6	146.2 (5)
O4—C51—C59—C40	178.7 (6)	Gd1—O5—Zn2—O6	−29.49 (15)
C28—C51—C59—C40	−2.1 (10)	C25—O5—Zn2—N3	6.6 (5)
C31—C40—C59—C51	−3.1 (11)	Gd1—O5—Zn2—N3	−169.00 (19)
C17—C41—C60—C52	4.3 (14)	C25—O5—Zn2—N4	79.1 (6)
C24—C52—C60—C41	−3.4 (14)	Gd1—O5—Zn2—N4	−96.5 (4)
C71—C64—C68—C69	−52.3 (15)	C25—O5—Zn2—Gd1	175.7 (5)
C64—C68—C69—C27	52.9 (14)	C21—O6—Zn2—O12	107.7 (5)
N2—C27—C69—C68	−176.6 (8)	Gd1—O6—Zn2—O12	−70.6 (2)
C45—C27—C69—C68	−54.6 (12)	C21—O6—Zn2—O5	−151.3 (5)
N1—C45—C71—C64	177.0 (8)	Gd1—O6—Zn2—O5	30.40 (16)
C27—C45—C71—C64	−57.6 (11)	C21—O6—Zn2—N3	−71.3 (6)
C68—C64—C71—C45	54.2 (13)	Gd1—O6—Zn2—N3	110.4 (3)
C42—C22—N1—C45	178.3 (7)	C21—O6—Zn2—N4	2.4 (5)
C42—C22—N1—Zn3	5.4 (11)	Gd1—O6—Zn2—N4	−176.0 (2)
C71—C45—N1—C22	7.3 (13)	C21—O6—Zn2—Gd1	178.3 (6)
C27—C45—N1—C22	−120.5 (8)	C23—N3—Zn2—O12	101.1 (6)
C71—C45—N1—Zn3	−178.4 (7)	C48—N3—Zn2—O12	−89.8 (5)
C27—C45—N1—Zn3	53.9 (7)	C23—N3—Zn2—O5	−4.3 (6)
C19—C26—N2—C27	−174.8 (7)	C48—N3—Zn2—O5	164.9 (5)
C19—C26—N2—Zn3	−6.9 (11)	C23—N3—Zn2—O6	−79.9 (7)
C69—C27—N2—C26	−55.9 (11)	C48—N3—Zn2—O6	89.2 (5)
C45—C27—N2—C26	−178.0 (7)	C23—N3—Zn2—N4	−156.5 (6)
C69—C27—N2—Zn3	134.9 (7)	C48—N3—Zn2—N4	12.7 (5)
C45—C27—N2—Zn3	12.7 (9)	C23—N3—Zn2—Gd1	−15.2 (7)
C17—C23—N3—C48	−170.2 (7)	C48—N3—Zn2—Gd1	154.0 (4)
C17—C23—N3—Zn2	−1.9 (11)	C32—N4—Zn2—O12	−114.3 (7)
C47—C48—N3—C23	−178.9 (7)	C47—N4—Zn2—O12	75.2 (5)
C35—C48—N3—C23	−50.6 (10)	C32—N4—Zn2—O5	60.8 (8)
C47—C48—N3—Zn2	11.7 (8)	C47—N4—Zn2—O5	−109.7 (5)
C35—C48—N3—Zn2	139.9 (6)	C32—N4—Zn2—O6	−4.1 (7)
C29—C32—N4—C47	175.8 (7)	C47—N4—Zn2—O6	−174.7 (5)
C29—C32—N4—Zn2	6.7 (12)	C32—N4—Zn2—N3	136.0 (7)
C58—C47—N4—C32	8.7 (12)	C47—N4—Zn2—N3	−34.5 (5)
C48—C47—N4—C32	−121.3 (8)	C32—N4—Zn2—Gd1	−7.8 (8)
C58—C47—N4—Zn2	179.8 (6)	C47—N4—Zn2—Gd1	−178.3 (4)
C48—C47—N4—Zn2	49.9 (7)	O1—Gd1—Zn2—O12	−161.5 (2)
C20—C15—O1—Zn3	176.8 (4)	O6—Gd1—Zn2—O12	117.4 (2)
C42—C15—O1—Zn3	−2.2 (8)	O11—Gd1—Zn2—O12	0.70 (18)
C20—C15—O1—Gd1	−8.2 (8)	O10—Gd1—Zn2—O12	−43.91 (17)
C42—C15—O1—Gd1	172.9 (4)	O5—Gd1—Zn2—O12	−107.7 (2)
O6—Gd1—O1—C15	47.8 (5)	O2—Gd1—Zn2—O12	55.5 (2)
O11—Gd1—O1—C15	−148.3 (4)	O3—Gd1—Zn2—O12	−170.07 (16)
O10—Gd1—O1—C15	−128.4 (5)	O8—Gd1—Zn2—O12	122.05 (16)
O5—Gd1—O1—C15	−34.6 (5)	O7—Gd1—Zn2—O12	−105.94 (16)
O2—Gd1—O1—C15	155.2 (5)	O1—Gd1—Zn2—O5	−53.8 (3)
O3—Gd1—O1—C15	8.6 (4)	O6—Gd1—Zn2—O5	−134.9 (2)
O8—Gd1—O1—C15	81.1 (5)	O11—Gd1—Zn2—O5	108.4 (2)
O7—Gd1—O1—C15	−63.3 (5)	O10—Gd1—Zn2—O5	63.8 (2)
Zn2—Gd1—O1—C15	−1.1 (6)	O2—Gd1—Zn2—O5	163.2 (2)
Zn3—Gd1—O1—C15	−176.1 (6)	O3—Gd1—Zn2—O5	−62.39 (19)
O6—Gd1—O1—Zn3	−136.08 (16)	O8—Gd1—Zn2—O5	−130.27 (19)
O11—Gd1—O1—Zn3	27.8 (4)	O7—Gd1—Zn2—O5	1.75 (18)
O10—Gd1—O1—Zn3	47.67 (17)	O1—Gd1—Zn2—O6	81.1 (3)
O5—Gd1—O1—Zn3	141.52 (15)	O11—Gd1—Zn2—O6	−116.7 (2)
O2—Gd1—O1—Zn3	−28.66 (15)	O10—Gd1—Zn2—O6	−161.3 (2)
O3—Gd1—O1—Zn3	−175.2 (2)	O5—Gd1—Zn2—O6	134.9 (2)
O8—Gd1—O1—Zn3	−102.74 (18)	O2—Gd1—Zn2—O6	−61.9 (2)
O7—Gd1—O1—Zn3	112.81 (19)	O3—Gd1—Zn2—O6	72.5 (2)
Zn2—Gd1—O1—Zn3	174.96 (5)	O8—Gd1—Zn2—O6	4.6 (2)
C51—C28—O2—Zn3	174.1 (4)	O7—Gd1—Zn2—O6	136.64 (19)
C19—C28—O2—Zn3	−6.9 (9)	O1—Gd1—Zn2—N3	−38.2 (3)
C51—C28—O2—Gd1	−5.0 (9)	O6—Gd1—Zn2—N3	−119.3 (3)
C19—C28—O2—Gd1	173.9 (4)	O11—Gd1—Zn2—N3	124.0 (3)
O1—Gd1—O2—C28	−152.0 (6)	O10—Gd1—Zn2—N3	79.4 (2)
O6—Gd1—O2—C28	−29.3 (6)	O5—Gd1—Zn2—N3	15.6 (3)
O11—Gd1—O2—C28	51.8 (6)	O2—Gd1—Zn2—N3	178.8 (3)
O10—Gd1—O2—C28	121.7 (6)	O3—Gd1—Zn2—N3	−46.8 (2)
O5—Gd1—O2—C28	85.6 (9)	O8—Gd1—Zn2—N3	−114.6 (2)
O3—Gd1—O2—C28	−119.0 (5)	O7—Gd1—Zn2—N3	17.4 (2)
O8—Gd1—O2—C28	−68.1 (5)	O1—Gd1—Zn2—N4	86.5 (3)
O7—Gd1—O2—C28	165.3 (5)	O6—Gd1—Zn2—N4	5.4 (3)
Zn2—Gd1—O2—C28	7.1 (6)	O11—Gd1—Zn2—N4	−111.3 (3)
Zn3—Gd1—O2—C28	179.3 (7)	O10—Gd1—Zn2—N4	−155.9 (2)
O1—Gd1—O2—Zn3	28.65 (16)	O5—Gd1—Zn2—N4	140.3 (3)
O6—Gd1—O2—Zn3	151.40 (15)	O2—Gd1—Zn2—N4	−56.5 (3)
O11—Gd1—O2—Zn3	−127.51 (17)	O3—Gd1—Zn2—N4	77.9 (2)
O10—Gd1—O2—Zn3	−57.60 (17)	O8—Gd1—Zn2—N4	10.0 (2)
O5—Gd1—O2—Zn3	−93.8 (7)	O7—Gd1—Zn2—N4	142.0 (2)
O3—Gd1—O2—Zn3	61.7 (2)	C30—O9—Zn3—O2	−42.1 (5)
O8—Gd1—O2—Zn3	112.56 (19)	C30—O9—Zn3—O1	42.1 (5)
O7—Gd1—O2—Zn3	−14.0 (2)	C30—O9—Zn3—N1	136.8 (5)
Zn2—Gd1—O2—Zn3	−172.17 (4)	C30—O9—Zn3—N2	−137.7 (4)
C44—C20—O3—C33	−0.8 (9)	C30—O9—Zn3—Gd1	2.9 (4)
C15—C20—O3—C33	180.0 (6)	C28—O2—Zn3—O9	−98.8 (5)
C44—C20—O3—Gd1	−173.2 (5)	Gd1—O2—Zn3—O9	80.60 (19)
C15—C20—O3—Gd1	7.6 (6)	C28—O2—Zn3—O1	149.9 (5)
O1—Gd1—O3—C20	−8.0 (4)	Gd1—O2—Zn3—O1	−30.69 (16)
O6—Gd1—O3—C20	−157.7 (4)	C28—O2—Zn3—N1	83.4 (6)
O11—Gd1—O3—C20	153.1 (4)	Gd1—O2—Zn3—N1	−97.2 (4)
O10—Gd1—O3—C20	45.3 (4)	C28—O2—Zn3—N2	9.8 (5)
O5—Gd1—O3—C20	132.7 (4)	Gd1—O2—Zn3—N2	−170.8 (2)
O2—Gd1—O3—C20	−42.8 (4)	C28—O2—Zn3—Gd1	−179.4 (6)
O8—Gd1—O3—C20	−94.5 (4)	C15—O1—Zn3—O9	108.4 (5)
O7—Gd1—O3—C20	70.5 (4)	Gd1—O1—Zn3—O9	−67.8 (2)
Zn2—Gd1—O3—C20	166.7 (4)	C15—O1—Zn3—O2	−151.4 (5)
Zn3—Gd1—O3—C20	−10.9 (4)	Gd1—O1—Zn3—O2	32.39 (17)
O1—Gd1—O3—C33	−179.8 (5)	C15—O1—Zn3—N1	2.1 (5)
O6—Gd1—O3—C33	30.6 (5)	Gd1—O1—Zn3—N1	−174.0 (2)
O11—Gd1—O3—C33	−18.7 (6)	C15—O1—Zn3—N2	−71.9 (6)
O10—Gd1—O3—C33	−126.5 (5)	Gd1—O1—Zn3—N2	112.0 (3)
O5—Gd1—O3—C33	−39.1 (5)	C15—O1—Zn3—Gd1	176.2 (6)
O2—Gd1—O3—C33	145.4 (5)	C22—N1—Zn3—O9	−117.0 (7)
O8—Gd1—O3—C33	93.7 (5)	C45—N1—Zn3—O9	68.9 (5)
O7—Gd1—O3—C33	−101.3 (5)	C22—N1—Zn3—O2	60.8 (8)
Zn2—Gd1—O3—C33	−5.1 (5)	C45—N1—Zn3—O2	−113.3 (6)
Zn3—Gd1—O3—C33	177.4 (5)	C22—N1—Zn3—O1	−3.7 (7)
C59—C51—O4—C54	1.6 (10)	C45—N1—Zn3—O1	−177.9 (5)
C28—C51—O4—C54	−177.7 (6)	C22—N1—Zn3—N2	136.9 (7)
C17—C25—O5—Zn2	−3.1 (8)	C45—N1—Zn3—N2	−37.2 (5)
C24—C25—O5—Zn2	178.0 (4)	C22—N1—Zn3—Gd1	−9.1 (8)
C17—C25—O5—Gd1	170.7 (4)	C45—N1—Zn3—Gd1	176.8 (4)
C24—C25—O5—Gd1	−8.1 (8)	C26—N2—Zn3—O9	101.2 (6)
O1—Gd1—O5—C25	−24.8 (5)	C27—N2—Zn3—O9	−90.2 (6)
O6—Gd1—O5—C25	−147.6 (5)	C26—N2—Zn3—O2	−3.1 (7)
O11—Gd1—O5—C25	128.0 (5)	C27—N2—Zn3—O2	165.5 (5)
O10—Gd1—O5—C25	58.3 (5)	C26—N2—Zn3—O1	−78.5 (7)
O2—Gd1—O5—C25	93.2 (9)	C27—N2—Zn3—O1	90.1 (6)
O3—Gd1—O5—C25	−64.0 (5)	C26—N2—Zn3—N1	−155.0 (7)
O8—Gd1—O5—C25	−115.2 (5)	C27—N2—Zn3—N1	13.6 (6)
O7—Gd1—O5—C25	7.1 (5)	C26—N2—Zn3—Gd1	−12.6 (8)
Zn2—Gd1—O5—C25	−174.9 (6)	C27—N2—Zn3—Gd1	156.0 (5)
Zn3—Gd1—O5—C25	13.1 (6)	O1—Gd1—Zn3—O9	121.4 (2)
O1—Gd1—O5—Zn2	150.10 (15)	O6—Gd1—Zn3—O9	−156.8 (2)
O6—Gd1—O5—Zn2	27.30 (15)	O11—Gd1—Zn3—O9	−44.04 (18)
O11—Gd1—O5—Zn2	−57.05 (16)	O10—Gd1—Zn3—O9	0.79 (18)
O10—Gd1—O5—Zn2	−126.74 (16)	O5—Gd1—Zn3—O9	57.0 (2)
O2—Gd1—O5—Zn2	−91.9 (7)	O2—Gd1—Zn3—O9	−106.0 (2)
O3—Gd1—O5—Zn2	110.87 (18)	O3—Gd1—Zn3—O9	125.58 (16)
O8—Gd1—O5—Zn2	59.71 (19)	O8—Gd1—Zn3—O9	−166.57 (16)
O7—Gd1—O5—Zn2	−177.9 (2)	O7—Gd1—Zn3—O9	62.04 (16)
Zn3—Gd1—O5—Zn2	−172.02 (4)	O1—Gd1—Zn3—O2	−132.6 (2)
C34—C21—O6—Zn2	176.5 (4)	O6—Gd1—Zn3—O2	−50.8 (3)
C29—C21—O6—Zn2	−3.1 (9)	O11—Gd1—Zn3—O2	62.0 (2)
C34—C21—O6—Gd1	−5.7 (8)	O10—Gd1—Zn3—O2	106.8 (2)
C29—C21—O6—Gd1	174.7 (5)	O5—Gd1—Zn3—O2	163.0 (2)
O1—Gd1—O6—C21	45.0 (5)	O3—Gd1—Zn3—O2	−128.4 (2)
O11—Gd1—O6—C21	−127.2 (5)	O8—Gd1—Zn3—O2	−60.6 (2)
O10—Gd1—O6—C21	−142.7 (5)	O7—Gd1—Zn3—O2	168.0 (2)
O5—Gd1—O6—C21	154.3 (5)	O6—Gd1—Zn3—O1	81.8 (3)
O2—Gd1—O6—C21	−35.7 (5)	O11—Gd1—Zn3—O1	−165.4 (2)
O3—Gd1—O6—C21	79.4 (5)	O10—Gd1—Zn3—O1	−120.6 (2)
O8—Gd1—O6—C21	7.0 (5)	O5—Gd1—Zn3—O1	−64.3 (2)
O7—Gd1—O6—C21	129.9 (5)	O2—Gd1—Zn3—O1	132.6 (2)
Zn2—Gd1—O6—C21	−178.3 (6)	O3—Gd1—Zn3—O1	4.2 (2)
Zn3—Gd1—O6—C21	−4.2 (6)	O8—Gd1—Zn3—O1	72.0 (2)
O1—Gd1—O6—Zn2	−136.78 (16)	O7—Gd1—Zn3—O1	−59.3 (2)
O11—Gd1—O6—Zn2	51.05 (17)	O1—Gd1—Zn3—N1	7.9 (3)
O10—Gd1—O6—Zn2	35.6 (4)	O6—Gd1—Zn3—N1	89.7 (3)
O5—Gd1—O6—Zn2	−27.41 (15)	O11—Gd1—Zn3—N1	−157.5 (3)
O2—Gd1—O6—Zn2	142.55 (15)	O10—Gd1—Zn3—N1	−112.7 (3)
O3—Gd1—O6—Zn2	−102.29 (17)	O5—Gd1—Zn3—N1	−56.5 (3)
O8—Gd1—O6—Zn2	−174.7 (2)	O2—Gd1—Zn3—N1	140.5 (3)
O7—Gd1—O6—Zn2	−51.8 (2)	O3—Gd1—Zn3—N1	12.1 (3)
Zn3—Gd1—O6—Zn2	174.04 (4)	O8—Gd1—Zn3—N1	79.9 (3)
C52—C24—O7—C37	9.7 (10)	O7—Gd1—Zn3—N1	−51.5 (3)
C25—C24—O7—C37	−169.5 (6)	O1—Gd1—Zn3—N2	−119.3 (3)
C52—C24—O7—Gd1	−177.5 (6)	O6—Gd1—Zn3—N2	−37.5 (3)
C25—C24—O7—Gd1	3.4 (7)	O11—Gd1—Zn3—N2	75.3 (3)
O1—Gd1—O7—C24	145.4 (4)	O10—Gd1—Zn3—N2	120.1 (3)
O6—Gd1—O7—C24	22.0 (4)	O5—Gd1—Zn3—N2	176.4 (3)
O11—Gd1—O7—C24	−64.6 (4)	O2—Gd1—Zn3—N2	13.3 (3)
O10—Gd1—O7—C24	−125.1 (4)	O3—Gd1—Zn3—N2	−115.1 (3)
O5—Gd1—O7—C24	−4.7 (4)	O8—Gd1—Zn3—N2	−47.2 (3)
O2—Gd1—O7—C24	−172.8 (4)	O7—Gd1—Zn3—N2	−178.6 (3)
